# Realising higher capacity and stability for disordered rocksalt oxyfluoride cathode materials for Li ion batteries†

**DOI:** 10.1039/d3ra05684h

**Published:** 2023-10-09

**Authors:** Ying Chen, Chun Huang

**Affiliations:** a Department of Materials, Imperial College London London SW7 2AZ UK; b The Faraday Institution Quad One, Becquerel Ave, Harwell Campus Didcot OX11 0RA UK; c Research Complex at Harwell, Rutherford Appleton Laboratory Didcot OX11 0FA UK a.huang@imperial.ac.uk

## Abstract

Disordered rocksalt (DRX) materials are an emerging class of cathode materials for Li ion batteries. Their advantages include better sustainability through wider choices of transition metal (TM) elements in the materials and higher theoretical capacities due to the redox reaction contributions from both the TM and O elements compared with state-of-the-art cathode materials. However, the realisable capacities of the DRX materials need to be improved as their charge transport kinetics and cycling stability are still poor. Here, Li_1.2_Mn_0.4_Ti_0.4_O_2_ (LMTO) and Li_1.3_Mn_0.4_Ti_0.3_O_1.7_F_0.3_ (LMTOF) are synthesised with abundant TMs of Mn and Ti only. Three approaches of partial substitution of O with F, reducing particle size and C coating on the particle surface are used simultaneously to improve realisable capacity, rate capability and stability. We rationalise that the improved electrochemical performance is due to the improved short and long range Li^+^ diffusion kinetics, electrical conductivity and reduced O loss. These strategies can also be applicable to a variety of DRX materials to improve performance.

## Abstract 2

Disordered rocksalt material containing abundant transition metal elements only and C coating with improved capacity, rate capability and cycling stability for battery cathodes.

## Graphical abstract

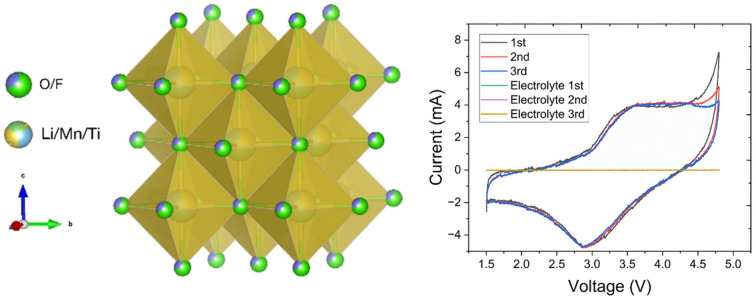

## 1 Introduction

Electrochemical energy storage systems such as Li ion batteries (LIBs) are attractive for electric transportation and storing electrical energy generated by intermittent renewable sources.^1^ The ever-growing demand on these applications requires LIBs to achieve higher capacities, lower costs and longer cycle lives. Within the LIB cell, the cathode material is one of the main factors that limits the capacity and dominates the battery costs.^2^ Two prevalent cathode materials, LiNi_0.8_Co_0.15_Al_0.05_O_2_ (NCA)^3^ and LiNi_*x*_Mn_*y*_Co_*z*_O_2_ (NMC),^4^ adopt a layered crystal structure that has an ordered arrangement of Li and transition metal (TM) atoms where Li^+^ ions diffuse in the alkali layer with a 2D pathway upon battery (dis)charge.^5^ Another widely used cathode material LiFePO_4_ (LFP) has an olivine crystal structure that still exhibits a clear 1D Li^+^ diffusion pathway.^6^ For decades, cation disordering, *i.e.,* mixing of the Li and transition metals (TMs) atoms in the lattice sites, has been considered to limit Li^+^ ion diffusion.^1^

Pioneering work demonstrated the feasibility of a disordered rocksalt (DRX) crystal structure for cathode materials Li_*x*_TM_2−*x*_O_2_ (0 ≤ *x* ≤ 2)^7^ that can offer high specific capacities of >300 mA h g^−1^ at 10 mA g^−1^ at 50 °C and energy densities of ∼1000 W h kg^−1^ in the voltage range of 1.5–4.8 V.^8^ It was found that Li^+^ ions are able to diffuse in the DRX crystal structure through a mechanism called ‘o–t–o diffusion’, which means the Li^+^ located in the octahedral (o) site diffuses to another octahedral (o) site *via* a tetrahedral (t) site.^9^ There are five types of Li^+^ ion diffusion environments in DRXs according to different cations mismatches: 0-TM, 1-TM, 2-TM, 3-TM and 4-TM local environments which correspond to Li_4_, Li_3_TM, Li_2_TM_2_, LiTM_3_ and TM_4_ tetrahedral clusters.^9^ However, only two types of the o–t–o channel allow efficient Li^+^ ion diffusion: 0-TM and 1-TM. The energy barrier of 1-TM is higher than that of 0-TM as the octahedral site in 1-TM is occupied by TM, which has a stronger electrostatic repulsion against Li^+^, reducing the mobility of Li^+^ ions. Therefore, effective Li^+^ diffusion in DRXs is mainly through the 0-TM channel with a diffusion barrier that is almost independent of the average tetrahedron size and the charge of face-sharing species.^9^ Monte Carlo simulations show that Li-rich cathode materials can achieve the interconnected 0-TM channels and form microscopic diffusion pathways.^9^ This mechanism has provided valuable insights for the design of DRX materials with Li^+^ percolation.^10^

Most studied chemical compositions of DRX materials include Li_1.25_Nb_0.25_Mn_0.5_O_2_,^11^ Li_1.2_Ti_0.4_Fe_0.4_O_2_,^11^ Li_1.2_Ti_0.4_Mn_0.4_O_2_ ^12^ and Li_1.25_Nb_0.25_V_0.5_O_2_.^13^ Almost all of these DRXs contain a type of TMs such as Ni, Mn and V with an unfilled outer shell electron orbital, and another type of d^0^ elements that are TM elements with a filled outer shell electron orbital such as Ti^4+^, Nb^5+^, Ru^5+^, Mo^6+^.^14–19^ The TMs with an unfilled outer shell electron orbital are redox centres that contribute to capacities, whereas the d^0^ elements do not contribute to the overall capacity, but play a crucial role in stabilising the crystal structures to accommodate distorted lattice sites due to their smaller sizes and higher charges. DRX materials present flexible compositional space, their stoichiometry is not limited to the explored compositions so far. The high capacities of DRXs reply on redox reactions of both TM and O in the cathode materials.^7,20,21^ On the other hand, O redox leads to irreversible O loss, structural changes, and ultimately poor capacity retention.^7,20,22^ Previous studies on the DRX materials have performed detailed simulations^23,24^ and characterisation such as *operando* X-ray total scattering and X-ray absorption spectroscopy-based techniques to study the changes of structures and oxidation states of TM.^25,26^ These studies have identified that the challenges of current DRX cathodes include limited realisable capacities due to restricted Li^+^ diffusion in both short and long ranges, limited electrical conductivity and rapid capacity losses of nearly 40% after just 30 cycles.^7,27^ As a result, these limitations hinder the commercial viability of the DRX materials.

**Fig. 1:**
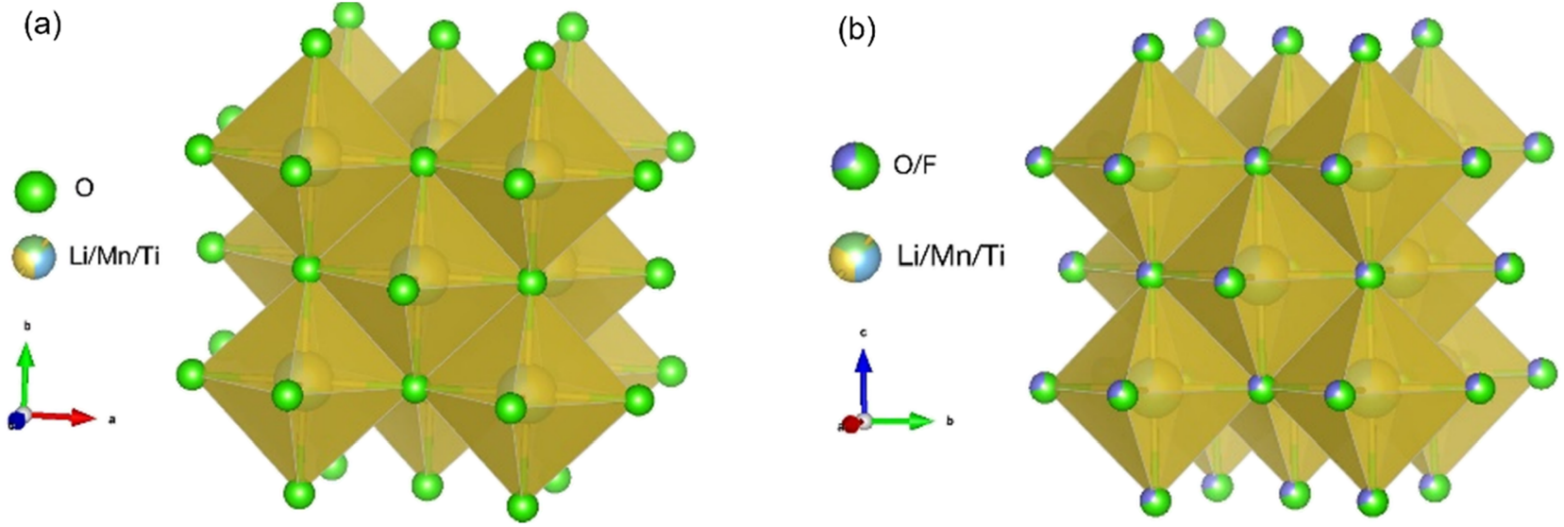
Cation disordered rocksalt (DRX) crystal structures of (a) Li_1.2_Mn_0.4_Ti_0.4_O_2_ (LMTO); and (b) Li_1.3_Mn_0.4_Ti_0.3_O_1.7_F_0.3_ (LMTOF).

Here, Mn as the redox centre and Ti as the d^0^ element are selected for the DRX materials as both Mn and Ti are significantly more abundant (0.1% and 0.6% of the Earth's crust, respectively)^28^ than Ni and Co (0.01% and 0.003% of the Earth's crust, respectively) present in the conventional layered cathode materials,^29^ and Ti has more favourable effects on Li^+^ percolation when coupled with Mn due to its size for the DRX materials.^1^ Three scalable experimental approaches of F substitution with O, reducing particle size and C coating on the particle surface are used to improve the electrochemical performance of the DRX materials. Replacing some of O^2−^ with F^−^ allows charge compensation with more freedom to tune the stoichiometry of cations such as minimising the level of redox inactive Ti^4+^ and increasing the Li^+^ content to increase capacity.^30^ Although there have been studies on substituting O with F,^31^ the amount of substitution is usually relatively low (F stoichiometry usually ≤0.2), *e.g.,* Li_1.2_Mn_0.55_Ti_0.25_O_1.85_F_0.15_.^32^ There have also been studies on F substitution for phosphate-based Na ion batteries Na_4_MnCr(PO_4_)_3_.^33^ Here, we synthesise Li_1.2_Mn_0.4_Ti_0.4_O_2_ (LMTO), the novelty of this work is to increase both Li and F amounts and synthesise Li_1.3_Mn_0.4_Ti_0.3_O_1.7_F_0.3_ (LMTOF). Fig. 1 compares the crystal structures of the synthesised LMTO and LMTOF, and post-synthesis treatments of reducing particle size and C coating are then performed on the synthesised materials. We systematically show the improvements of each approach on realisable capacities, rate capability and cycling stability. We rationalise the improved electrochemical performance is due to improved Li^+^ diffusion kinetics, electrical conductivity, and reduced irreversible O loss.

## 2 Results and discussion

### 2.1 Physical and chemical properties

Six types of cathode materials were prepared (see Experimental), as summarised in Table 1. Namely, as-synthesised LMTO (1) and LMTOF (2), post-synthesis ball milling to reduce particle size of each type of material for LMTO-B (3) and LMTOF-B (4), and post-synthesis ball milling with C to reduce particle size and add C coating on the particle surface simultaneously for each type of material for LMTO@C (5) and LMTOF@C (6).

**Table 1:** A summary of the six types of synthesised cathode materials and their post-synthesis treatment conditions

Sample	Materials	Post-synthesis ball milling	Carbon coating
1	LMTO	✗	✗
2	LMTOF	✗	✗
3	LMTO-B	✓	✗
4	LMTOF-B	✓	✗
5	LMTO@C	✓	✓
6	LMTOF@C	✓	✓

**Fig. 2:**
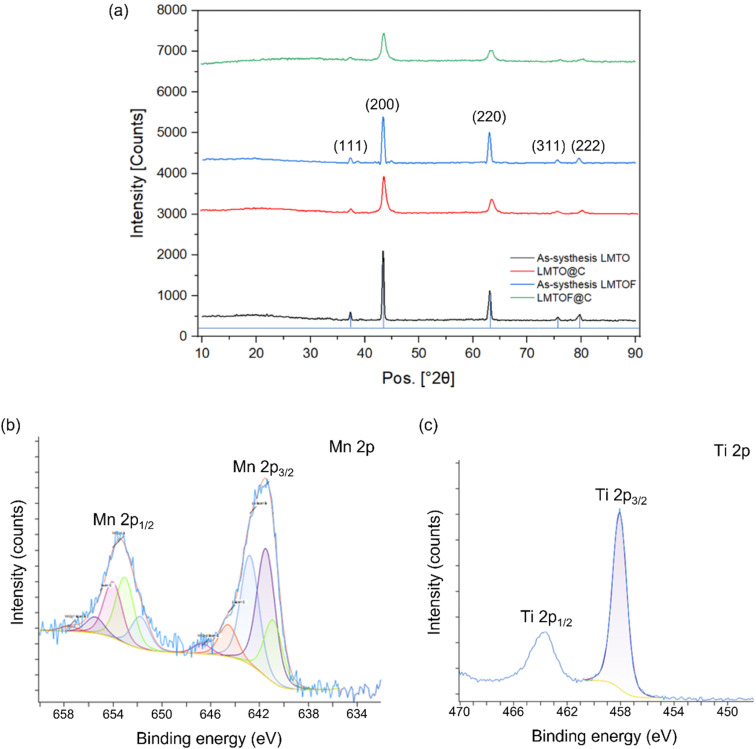
(a) XRD patterns of as-synthesised LMTO and LMTOF, and their C-coated equivalents; (b) XPS Mn2p spectra and (c) XPS Ti2p spectra of LMTOF.

Fig. 2a shows the X-ray diffraction (XRD) patterns of LMTO, LMTOF and their C coated samples. Rietveld refinement was performed^34,35^ and the lattice parameters of LMTO and LMTOF were 4.1703 Å and 4.1811 Å, respectively. Table S3† lists the crystallographic parameters of LMTO and LMTOF. Rietveld refinement was also used for other new cathode materials for Na ion batteries, *e.g.* NASICON Na_3_Cr_0.5_V_1.5_(PO_4_)_3_ (ref. 36) and Na4MnCr(PO_4_)_3_.^37^ The LMTO and LMTOF patterns corresponded well to the space group *Fm*3̄m, indicating single phase DRX materials were synthesised.^19^ The XRD patterns after C coating show the diffraction peaks at the same *2θ* positions, but the peaks were broadened compared with those before C coating, indicating the DRX crystal structure was maintained and the particle size was reduced. Inductively coupled plasma - atomic emission spectroscopy (ICP-AES) results (Tables S1 and S2 in the ESI†) confirmed the stoichiometries were Li : Mn : Ti = 1.2 : 0.4 : 0.4 and Li : Mn : Ti = 1.3 : 0.4 : 0.3 for the as-synthesised LMTO and LMTOF respectively. X-ray photoelectron spectroscopy (XPS) analysis was performed to study the oxidation states of Mn and Ti in LMTOF.^38^ The Mn_2p_ spectrum in Fig. 2b shows prominent peaks at 641.5 and 653.1 eV, which are the characteristic peaks of Mn 2p_3/2_ and 2p_1/2_, confirming Mn was predominantly in the 3+ oxidation state.^39^ The Ti_2p_ spectrum in Fig. 2c shows strong peaks at 458.1 and 463.7 eV, corresponding to the characteristic peaks of Ti 2p_3/2_ and Ti 2p_1/2_, confirming Ti was in the 4+ oxidation state.^40–42^ These results suggest that the oxidation states of Mn and Ti were consistent to the designed chemical states.

**Fig. 3:**
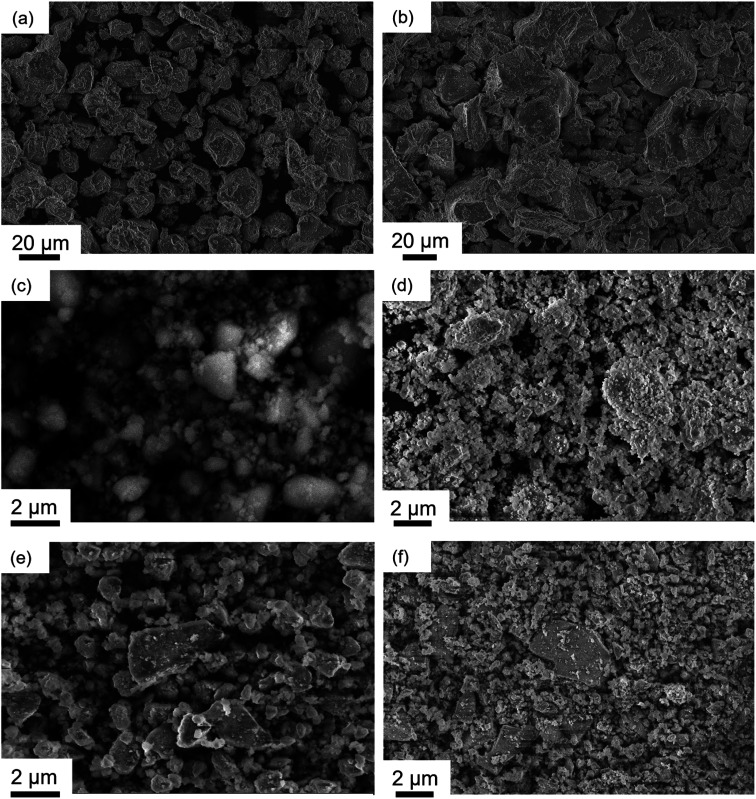
SEM images of (a) as-synthesised LMTO; (b) as-synthesised LMTOF; (c) LMTO-B; (d) LMTOF-B; (e) LMTO@C; and (f) LMTOF@C.

The scanning electron microscopy (SEM) images of as-synthesised LMTO and LMTOF in Fig. 3a and b show wide particle size distributions of 3–25 μm for LMTO and 6–40 μm for LMTOF, the as-synthesised particle size was consistent with those in the literature using similar synthesis methods.^7,20^ Fluorination led to particle growth, one of the reasons of using the two-stage sintering during the LMTOF synthesis was to prevent F from being released during heating.^30^ Ball-milling with and without C were then used to reduce the particle sizes. Fig. 3c–f show the SEM images of LMTO-B, LMTOF-B, LMTO@C and LMTOF@C, revealing significant reductions in the particle size to 500 nm to 4.5 μm and improvements in the particle size uniformity after post-synthesis treatment for all four types of samples, and C coating did not increase the particle size.

**Fig. 4:**
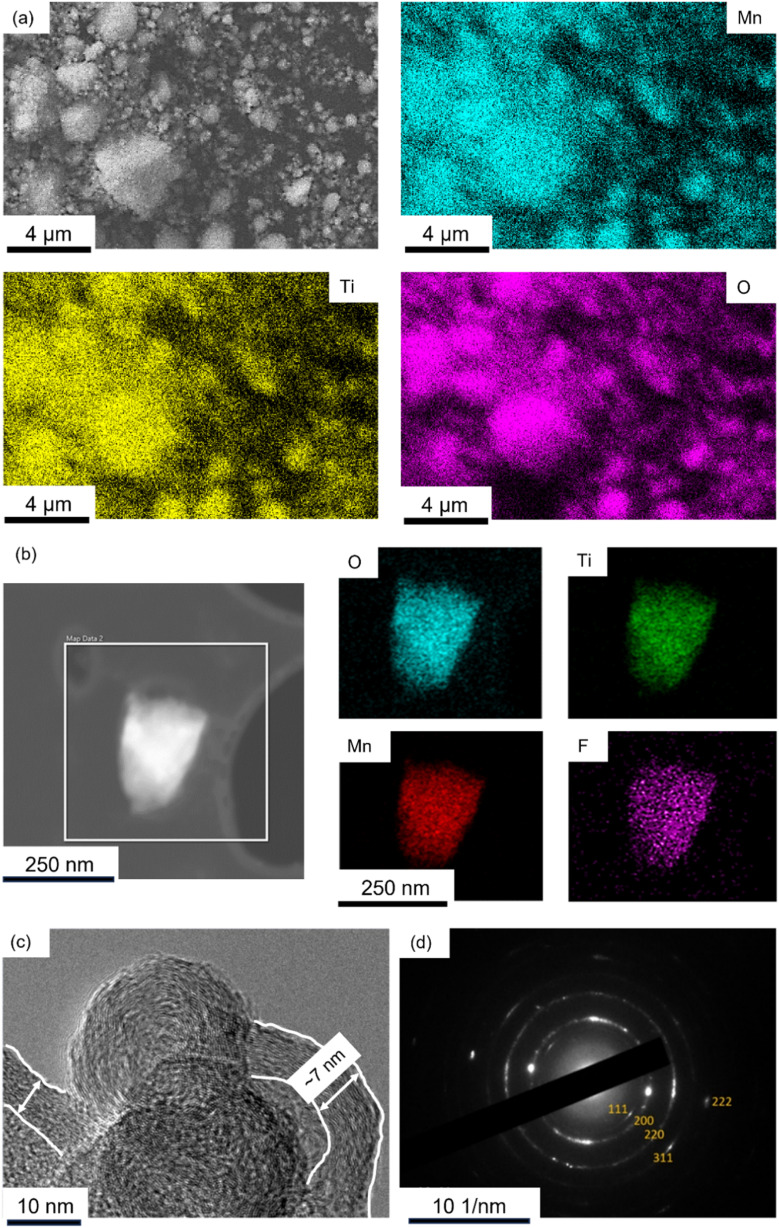
(a) SEM image of LMTOF@C and the corresponding EDS mapping of Mn, Ti and O; showing the TM elements are omogeneously distributed throughout the bulk powder. (b) TEM image of a single particle of LMTOF@C and the corresponding EDS mapping of O, Ti, Mn and F in STEM mode; showing the elements are also homogeneously distributed inside single particles. (c) TEM image of the edge of LMTOF@C particles showing C coating on the particle surface; and (d) electron diffraction pattern of the particles from (c) where each diffraction ring corresponds to the diffraction plane of the DRX crystal structure.

Fig. 4a is energy dispersive spectroscopy (EDS) elemental mapping of the SEM image of LMTOF@C (corresponding EDS spectrum in Fig. S1†), showing Mn and Ti were homogeneous throughout the bulk powder. Mn and Ti were also found to be homogeneous throughout the bulk powder of LMTOF-B in the SEM-EDS elemental mapping in Fig. S2.†Fig. 4b is a transmission electron microscopy (TEM) image of a single LMTOF@C particle and the corresponding EDS mappings in the scanning transmission electron microscopy (STEM) mode, confirming the distributions of Mn, Ti and F were also uniform inside an individual single particle. Additionally, Mn and Ti were found to be homogeneous inside single particles of LMTO@C without F substitution (Fig. S3†). Fig. 4c is a TEM image of the edge of LMTOF@C particles, showing graphitisation texture coating of ∼7 nm in thickness on the particle surface. The texture was similar to the C texture reported in the literature,^43^ indicating a thin layer of C was coated on the particles. Electron diffraction of the LMTOF@C particles in Fig. 4d shows each diffraction ring corresponded to each diffraction plane in the XRD patterns in Fig. 2a, again confirming the crystalline structure was maintained after the post-synthesis treatments.

### 2.2 Electrochemical properties

**Fig. 5:**
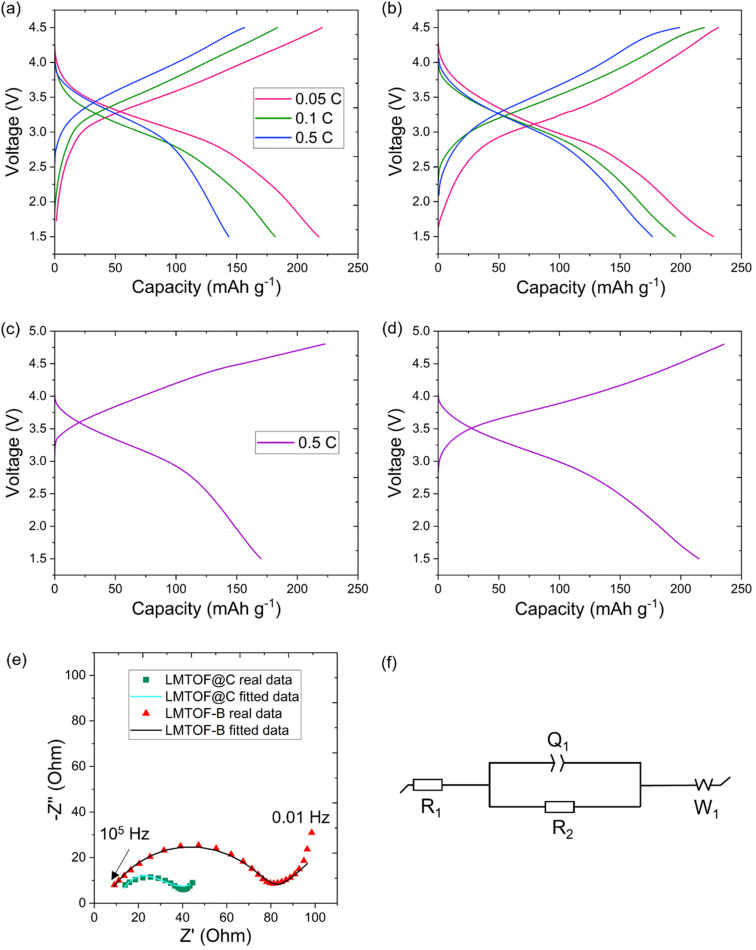
Charging and discharging profiles of (a) LMTO@C and (b) LMTOF@C at 1.5–4.5 V at 0.05, 0.1 and 0.5C; and (c) LMTO@C and (d) LMTOF@C at 1.5–4.8 V at 0.5C. (e) EIS plots of the prepared samples with equivalent circuit model of LMTOF-B and LMTOF@C.

The as-synthesised LMTO and LMTOF exhibited no capacity, which may be due to limited long range Li^+^ diffusion in the large-size (up to ∼40 μm) particles as the Li^+^ diffusion coefficient *D*_Li_ in Li-rich DRXs (10^−16^ to 10^−17^ cm^2^ s^−1^) is still lower than *D*_Li_ in the conventional layered cathode materials (10^−11^ to 10^−8^ cm^2^ s^−1^) at room temperature.^43–45^ To investigate the effects of F substitution on the O site, Fig. 5a and b show the galvanostatic (dis)charge profiles of LMTO@C and LMTOF@C at 0.05, 0.1 and 0.5C in the voltage window of 1.5–4.5 V. LMTOF@C exhibited higher reversible capacities than LMTO@C at all C rates. The capacity of LMTOF@C was 10 mA h g^−1^ higher than that of LMTO@C at 0.05C, and the difference increased to 33 mA h g^−1^ at 0.5C. The reversible capacity retention of LMTOF@C (81%) was higher than LMTO@C (65%) as the C rate increased, indicating a higher rate capability. These results can be attributed to (i) particle size reduction improved long range Li^+^ diffusion kinetics; and (ii) F substitution improved short range Li^+^ percolation kinetics because the more electronegative F^−^ exhibits a stronger bonding tendency with Li^+^ than O^2–^ (ref. 46) this maximises the Li^+^ content around F^−^, resulting in a greater number of Li-rich 0-TM clusters and improved Li^+^ percolation.

Fig. S3a and b† are the galvanostatic (dis)charge profiles of LMTO-B and LMTOF-B in the voltage window of 1.5–4.8 V at 0.5C, showing very low discharge capacities of 23 and 45 mA h g^−1^, respectively. The voltage window of 1.5–4.8 V was also used for other DRX cathode materials using the same electrolyte in the literature.^7,17,20,27^ After C coating was applied, the charge capacities were increased to 222 and 235 mA h g^−1^ and the discharge capacities were increased to 170 and 215 mA h g^−1^ for LMTO@C and LMTOF@C respectively in the same voltage window at 0.5C (Fig. 5c and d). Table 2 summarises the electrochemical performance of the four samples: LMTO-B, LMTOF-B, LMTO@C, and LMTOF@C. LMTOF@C exhibited the most favourable electrochemical performance, this observation strongly suggests the beneficial impact of both the carbon coating and fluorination of the cathode material. We then compared the performance of LMTOF@C with other similar composites in the literature (Table 3). The capacity of LMTOF@C at 0.5C was similar to ∼200 mA h g^−1^ achieved by a composition of Li_1.2_Mn_0.6_Ti_0.2_O_1.8_F_0.2_, but the latter used a significantly lower (dis)charge current of 30 mA g^−1^ (equivalent to ∼0.08C).^32^ The capacity of LMTOF@C was also competitive with 210 mA h g^−1^ at 0.1C achieved by Li_2_Mn_0.5_V_0.5_O_2_F (LMVOF),^47^ but again LMVOF was tested at 1/5th of the (dis)charge C rate compared with LMTOF@C. The comparisons show LMTOF@C exhibited faster Li^+^ percolation kinetics due to the higher amount of Li^+^ and electronegative F^−^. Electrochemical impedance spectroscopy (EIS) was used to further investigate the effects of C coating. Fig. 5e shows the Nyquist plots of LMTOF-B and LMTOF@C after 1 cycle of (dis)charge at 0.5C, the equivalent circuit model used to fit the EIS data is in Fig. 5f. Both Nyquist plots exhibited a semi-circle in the high frequency region and an inclined line at low frequency. *R*_1_ is the intercept of the Nyquist plot and the *Z*′ axis, and represents the internal resistance of the cell such as the ohmic resistance from electrolyte and other components.^48^*R*_2_ is the diameter of the semi-circle and relates to the charge transfer resistance of the electrode whereas *Q*_1_ is the constant phase element of the porous electrodes, and the linear part of the Nyquist plot is the Warburg diffusion impedance *W*_1_*.*^49–51^ Using the equivalent circuit model, *R*_1_ was calculated to be 2–4 Ω for both samples, LMTOF@C exhibited a significantly smaller *R*_2_ of 25.3 Ω compared with 75.4 Ω for LMTOF-B. The reduced charge transfer resistance of LMTOF@C suggests the C coating on the particle surface increased electronic conductivity of the material.

**Table 2:** A summary of the four types of synthesised cathode materials and their electrochemical performance at 0.5C

Materials	Capacity (mA h g^−1^)			
	Charge (1.5–4.5 V)	Discharge (1.5–4.5 V)	Charge (1.5–4.8 V)	Discharge (1.5–4.8 V)
LMTO-B	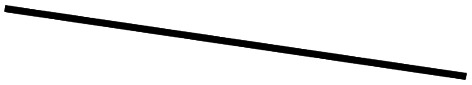	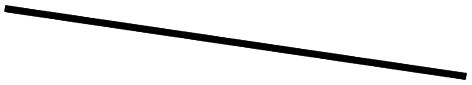	27	23
LMTOF-B	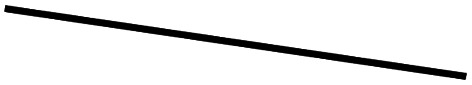	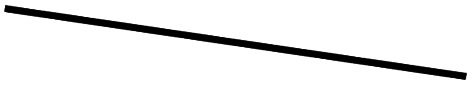	47	45
LMTO@C	152	148	222	170
LMTOF@C	199	177	235	215

**Table 3:** A summary of performance with similar composites

Materials	Discharge capacity (mA h g^−1^)	C rate (h^−1^)	Ref.
Li_1.3_Mn_0.4_Ti_0.3_O_1.7_F_0.3_ with C coating (LMTOF@C)	215	0.5	This work
Li_1.2_Mn_0.6_Ti_0.2_O_1.8_F_0.2_	200	0.08	32
Li_2_Mn_0.5_V_0.5_O_2_F	210	0.1	47

**Fig. 6:**
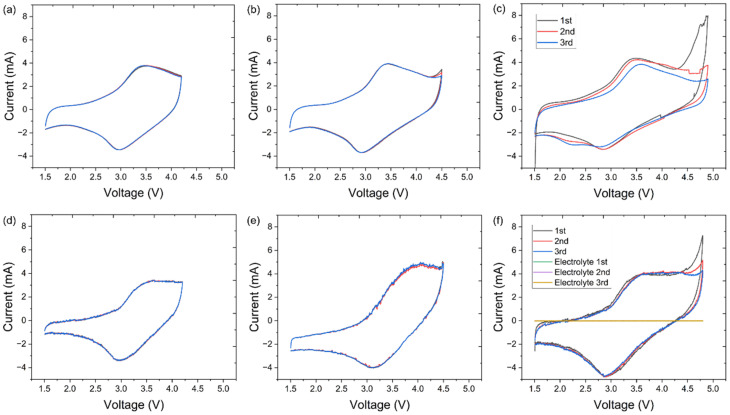
CV curves of LMTO@C at three different UCV of (a) 4.2 V; (b) 4.5 V; (c) 4.8 V. CV curves LMTOF@C at three different UCV of (d) 4.2 V; (e) 4.5 V; (f) 4.8 V. (f) also includes the CV curves of electrolyte in a symmetrical cell of SS spacer/electrolyte/SS spacer configuration. All tests were performed at 0.05 mV s^−1^ at the same conditions.

Cyclic voltammetry (CV) was performed to understand the cation and anion redox processes. Fig. 6a–c compares the CV scans for LMTO@C collected with a fixed lower cutoff voltage (LCV) and three different upper cutoff voltages (UCV): 4.2, 4.5 and 4.8 V at the first 3 cycles. When UCV was 4.2 V, an anodic peak at ∼3.5 V and a cathodic peak at ∼3.0 V were found, referring to the reversible Mn^3+^/Mn^4+^ redox reaction. Increasing UCV to 4.5 V triggered a new anodic peak at 4.5 V, indicating partial activation of O redox.^17^ During the cathodic scan, only one peak was observed, indicating the reductions of Mn and O were coupled. Further increasing UCV to 4.8 V largely increased the intensity of the O oxidation peak, indicating O redox contribution increased.^30,32,52^ A similar set of CV scans for LMTOF@C is shown in Fig. 6d–f, and the same phenomenon was observed when UCV was increased from 4.2 to 4.8 V. In addition, a CV scan was performed using a symmetrical cell of the stainless steel (SS) spacer/electrolyte/SS spacer configuration and the same electrolyte in the voltage range of 1.5–4.8 V at the testing conditions in the first few cycles (Fig. 6f). No peaks were observed in the CV scan, indicating the electrolyte did not undergo decomposition at the testing conditions during the first few cycles, in agreement with the literature.^30,53^ Hence, the peak at ≥4.5 V refers to O redox that contributes to the higher capacity of Li-rich DRX materials.^54^ The O redox peak intensity at ≥4.5 V reduced significantly at the 3rd cycle for LMTO@C due to oxygen loss. Although the same peak intensity also reduced, the current of the peak was still higher for LMTOF@C (4.31 A) than that of LMTO@C (2.59 A).

Fig. S5† shows the (dis)charge cycling performance of LMTO@C and LMTOF@C at 1.5–4.8 V at 0.5C. The capacity reduction was 55.4 mA h g^−1^ over 100 cycles for LMTOF@C (capacity retention 74%) whereas the capacity reduction was 84.2 mA h g^−1^ for LMTO@C (capacity retention 51%). The discharge capacity retention of LMTOF@C was also higher than other DRX oxides with similar Li stoichiometry and without F substitution, *e.g.,* 43% after 50 cycles for Li_1.3_Fe_0.4_Nb_0.3_O_2_ (ref. 2) and 52% for Li_1.2_Ti_0.4_Mn_0.4_O_2_ after 50 cycles.^30^ The results corroborate with the (dis)charge profiles in Fig. 5c and d that the capacity difference between charge and discharge was 52.7 mA h g^−1^ for LMTO@C which was reduced to 20.2 mA h g^−1^ for LMTOF@C, showing both higher capacity and reversibility for LMTOF@C than LMTO@C. The results suggest that fluorination suppresses O loss, while the oxidation state of lattice O is 2−, the oxidation state of F is 1−, so F substitution allows TM to have more d electrons by reduction.^55^ In general, the charge compensation for the Li^+^ ion extraction preferentially happens through d orbitals of TMs as they are closer to the Fermi level than O.^56^ As a result, F reduces O loss without inhibiting significantly redox reactions that contribute to capacities.

## 3 Conclusions

Li_1.2_Mn_0.4_Ti_0.4_O_2_ (LMTO) and Li_1.3_Mn_0.4_Ti_0.3_O_1.7_F_0.3_ (LMTOF) with abundant transition metal (TM) elements of Mn and Ti are synthesised. Three scalable experimental approaches of F substitution with O, reducing particle size and C coating on the particle surface were used together to improve the electrochemical performance of the disordered rocksalt (DRX) crystal structured cathode materials. The discharge capacity was increased from 45 to 215 mA h g^−1^ at 0.5C for LMTOF@C after using these approaches together. Furthermore, the capacity increase became higher as the (dis)charge rate increased, showing LMTOF@C also exhibited a higher rate capability. The capacity retention after 100 cycles at 0.5C was increased from 51% for LMTO@C to 74% for LMTOF@C. We rationalise the improved performance was due to improved Li^+^ diffusion kinetics in both short and long range, electrical conductivity and reduced irreversible O loss. In particular, the role of F was to maximise Li^+^ content around F^−^ to achieve a greater number of 0-TM clusters in the crystal structure for the short range Li^+^ percolation and to allow TM to undergo more redox reactions and suppress O loss.

## 4. Experimental

### 4.1 Synthesis methods

To synthesise Li_1.2_Mn_0.4_Ti_0.4_O_2_ (LMTO), stoichiometric amounts of Mn_2_O_3_ (99%; Sigma Aldrich), TiO_2_ (99%; Sigma Aldrich), Li_2_CO_3_ (99%; Sigma Aldrich) were thoroughly mixed by wet ball milling with ethanol for 24 h at 400 rpm. To synthesise Li_1.3_Mn_0.4_Ti_0.3_O_1.7_F_0.3_ (LMTOF), stoichiometric amounts of Mn_2_O_3_ (99%; Sigma Aldrich), TiO_2_ (99%; Sigma Aldrich), Li_2_CO_3_ (99%; Sigma Aldrich) and LiF (99%; Sigma Aldrich) were thoroughly mixed by wet ball milling with ethanol for 24 h at 400 rpm. The ball-milled mixtures were dried overnight in air and pressed into pellets at 5 tonnes for 5 min. The LMTO pellet was calcinated at 900 °C for 10 h in an Ar atmosphere tube furnace followed by natural cooling. The LMTOF pellets were heated at 600 °C for 3 h as a pre-calcination step followed by sintering at 1000 °C under for 6 h in an Ar atmosphere. The pre-calcination step for synthesising the fluorinated material LMTOF was to prevent the loss of reactive LiF starting material during sintering.^57^ All the samples were sintered at a ramp rate of 5 °C min^−1^. The annealed pellets were finally grounded into powder and stored in an Ar-filled glovebox until use. To prepare LMTO@C and LMTOF@C, the as-synthesised materials were mixed with 10 wt% conductive agent C65 using dry ball milling at 400 rpm for 12 h. For comparison, the as-synthesised LMTO and LMTOF powders were also ball milled at the same condition without C to make LMTO-B and LMTOF-B.

### 4.2 Characterisation methods

Phase purity of the synthesised materials was checked by X-ray diffraction (XRD) (Bruker AXS D2 Phaser) operating with Cu K-alpha in the range of 2*θ* range of 10–90°, step size of 0.02°, and a scan rate of 0.5° min^−1^. Rietveld refinement of XRD was performed using GSAS II software. Scanning electron microscopy (SEM) images were obtained on Zeiss Gemini Sigma 300 with an energy-dispersive X-ray spectroscopy (EDS) detector and JEOL JSM-6010PLUS/LA. EDS was also obtained from JEOL STEM 2100Plus. Transmission electron microscopy (TEM) was carried out on a JEOL-2100F microscope operating. X-ray photoelectron spectroscopy (XPS) was conducted on Thermo Scientific K-Alpha+ X-ray photoelectron spectrometer equipped with a MXR3 Al Kα monochromatic X-ray source (*hν* = 1486.6 eV). All spectra were first calibrated using the C_1s_ peak at 284.6 eV as a reference, the XPS peak fitting and analysis were performed using the standard Avantage software. Average compositions of samples were measured by inductively coupled plasma - atomic emission spectroscopy (ICP-AES) using a Thermo Scientific iCAP 6000 series instruments. Li, Mn and Ti calibration standard solutions (*Trace*CERT^@^) were diluted by water into 0, 5, 10, 15, and 20 ppm. Linear calibration curves were achieved for all elements. LMTO and LMTOF were dissolved in the high purity concentrated nitric acid (HNO_3_) (69%) first and then the solution were diluted with water for the ICP-AES characterisation.

To prepare the CR2032 type coin-cell, 70 wt% cathode active materials were mixed with 20 wt% conducting agent (C65), and 10 wt% polytetrafluoroethylene (PVDF, Sigma Aldrich). The mixture was dissolved in *N*-methyl-2-pyrrolidone solvent (NMP, Sigma Aldrich) and the slurry was cast using a doctor blade method on Al foils followed by drying at 85 °C for 2 h under vacuum. The cathode was then cut into disks with a diameter of 16 mm, the average electrode thickness was 200 μm. Coin cells were assembled using 1 M LiPF_6_ (in 1 : 1(v/v) ethylene carbonate (EC)–ethyl methyl carbonate (EMC)) as the electrolyte. Polyethylene membrane (Celgard 2500) and Li chip were used as the separator and anode. Coin cells were assembled in the Ar-filled glovebox and tested on Arbin cycling instruments in different voltage ranges and at different C rates. Cyclic voltammetry (CV) tests were performed between in different voltage ranges on BioLogic Science instruments. Electrochemical impedance spectroscopy (EIS) was performed using Biologic Science Instruments over the frequency range from 10 mHz to 100 kHz with a voltage amplitude of 10 mV. All tests were performed at room temperature.

## Conflicts of interest

There are no conflicts to declare.

## Supplementary Material

RA-013-D3RA05684H-s001
