# Supplementary material for: Realising higher capacity and stability for disordered rocksalt oxyfluoride cathode materials for Li ion batteries
Source: RSC Adv. 2023 Oct 9;13(42):29343–53. doi: 10.1039/d3ra05684h (PMC10560877; doi:10.1039/d3ra05684h)
Supplement: RA-013-D3RA05684H-s001 [file RA-013-D3RA05684H-s001.pdf]

Electronic Supplementary Material (ESI) for RSC Advances.
This journal is © The Royal Society of Chemistry 2023

Realising higher capacity and stability for disordered rocksalt oxyfluoride cathode materials for Li ion batteries

*Ying Chen[1] and Chun Huang[1,2,3]**

[1]Department of Materials, Imperial College London, London SW7 2AZ, UK

[2]The Faraday Institution, Quad One, Becquerel Ave, Harwell Campus, Didcot OX11 0RA, UK

[3]Research Complex at Harwell, Rutherford Appleton Laboratory, Didcot OX11 0FA, UK

Email: a.huang@imperial.ac.uk

Table S1: Results from ICP-AES for LMTOF.

LMTO 5% excess in Li_2CO_3			
Element	Li4602	Mn2576	Ti3349
Unit	ppm	ppm	ppm
Avg	8.723	23.03	20.07
Stddev	0.03	0.04	0.08
%RSD	0.2913	0.192	0.3895

Table S2: Results from ICP-AES for LMTOF.

LMTOF 20% excess in Li_2CO_3			
Element	Li4602	Mn2576	Ti3349
Unit	ppm	ppm	ppm
Avg	3.028	8.011	4.715
Stddev	0.054	0.019	0.039
%RSD	1.79	0.2336	0.8249

Table S3: Rietveld refinement for LMTO and LMTOF materials.

Materials	Atom	Site	X	Y	Z	fraction	Rwp	GOF (good of fitness)
$Li_{1.2}Mn_{0.4}Ti_{0.4}O_2$	Li	4a	0	0	0	0.6	0.0377	1.91
	Mn	4a	0	0	0	0.2		
	Ti	4a	0	0	0	0.2		
	O	4b	1/2	1/2	1/2	1		
$Li_{1.2}Mn_{0.4}Ti_{0.3}O_{1.7}F_{0.3}$	Li	4a	0	0	0	0.6	0.0535	2.27
	Mn	4a	0	0	0	0.2		
	Ti	4a	0	0	0	0.15		
	O	4b	1/2	1/2	1/2	0.85		
	F	4b	1/2	1/2	1/2	0.15		

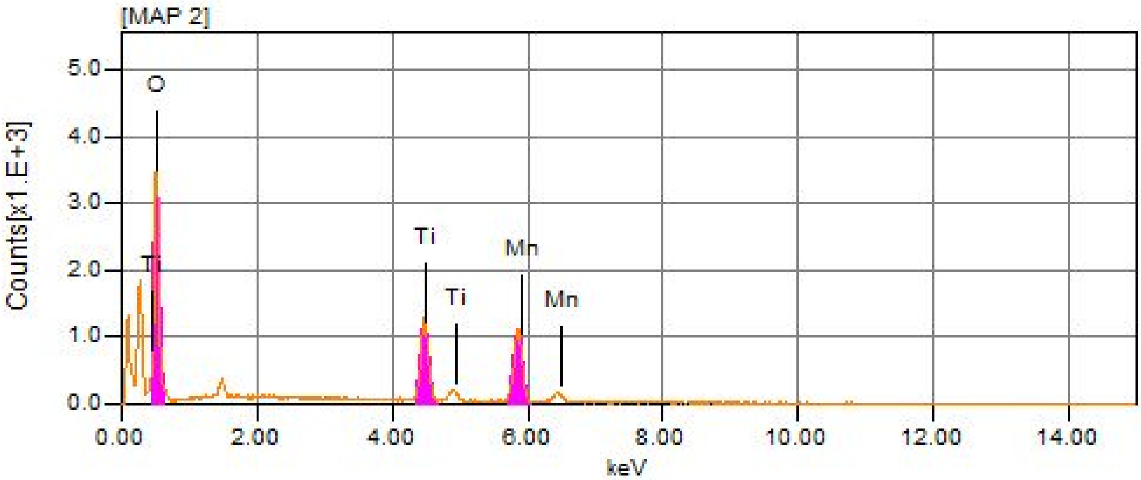

Figure S1: EDS spectrum for the EDS elemental mapping of the SEM image of LMTOF@C, showing the presence of Ti and Mn.

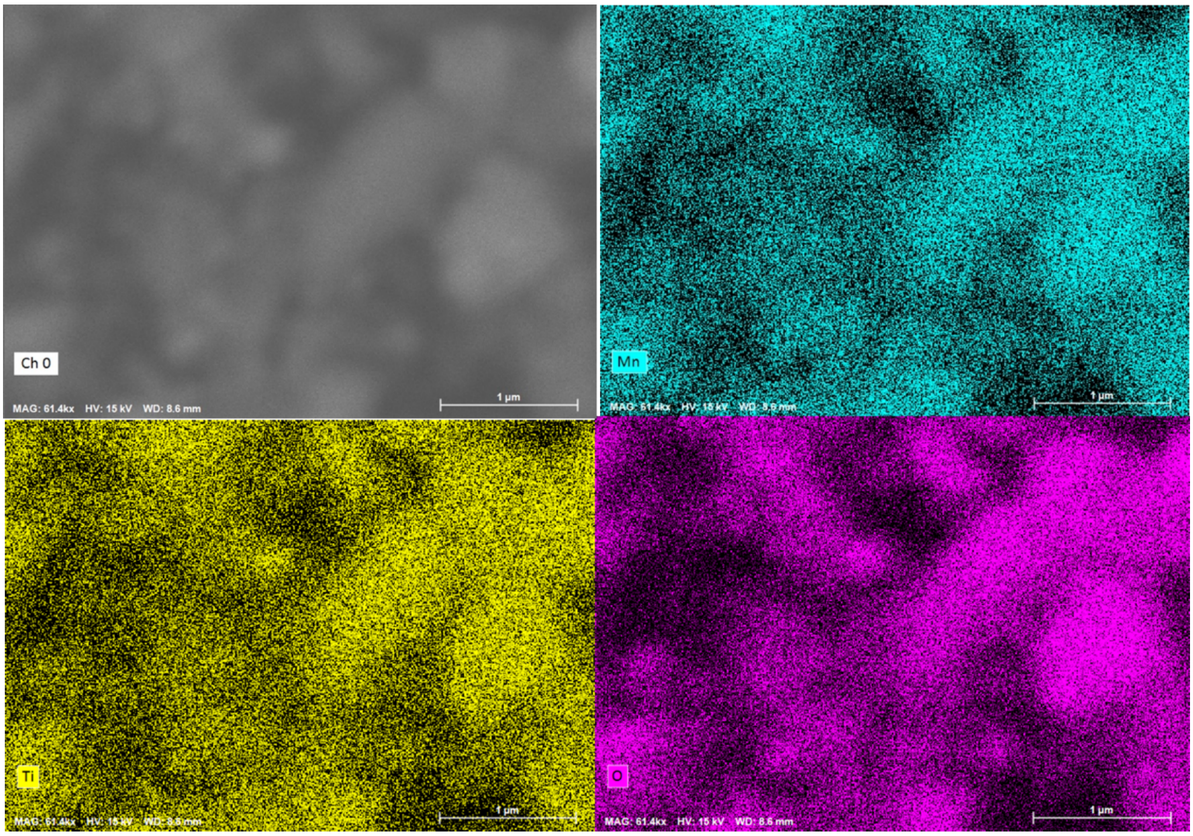

Figure S2: SEM image of LMTO@C and the corresponding EDS mapping of Mn, Ti and O.

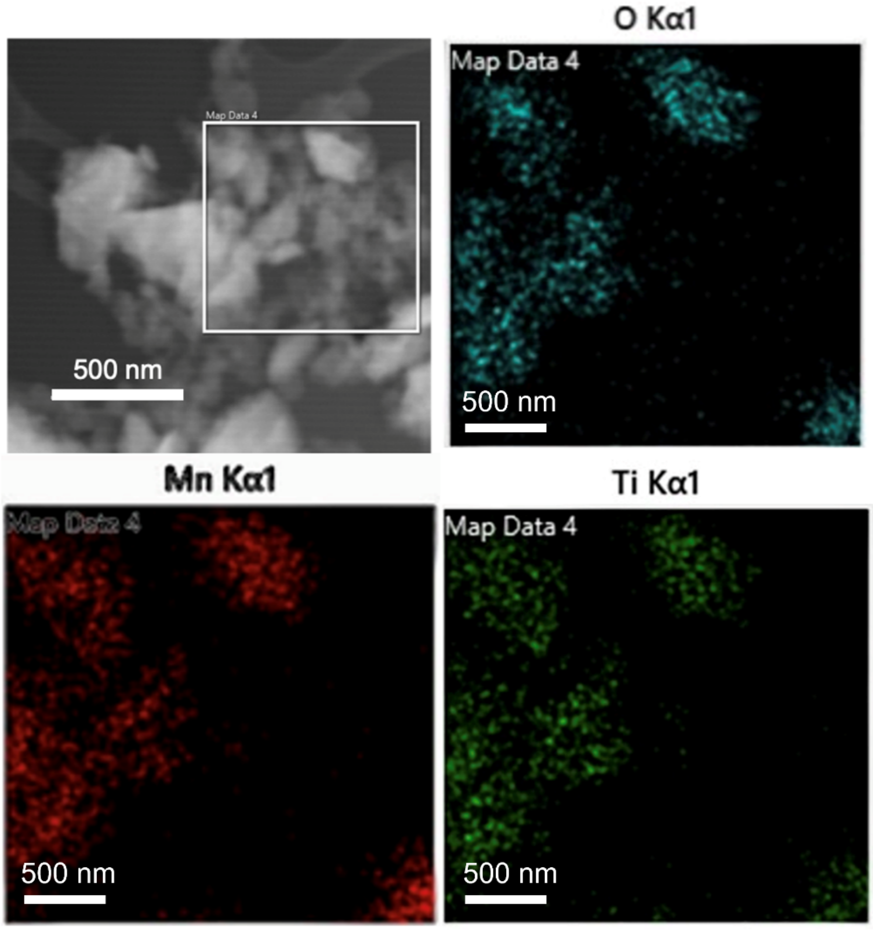

Figure S3: TEM image of individual particles of LMTO@C and the corresponding EDS mapping of the particles.

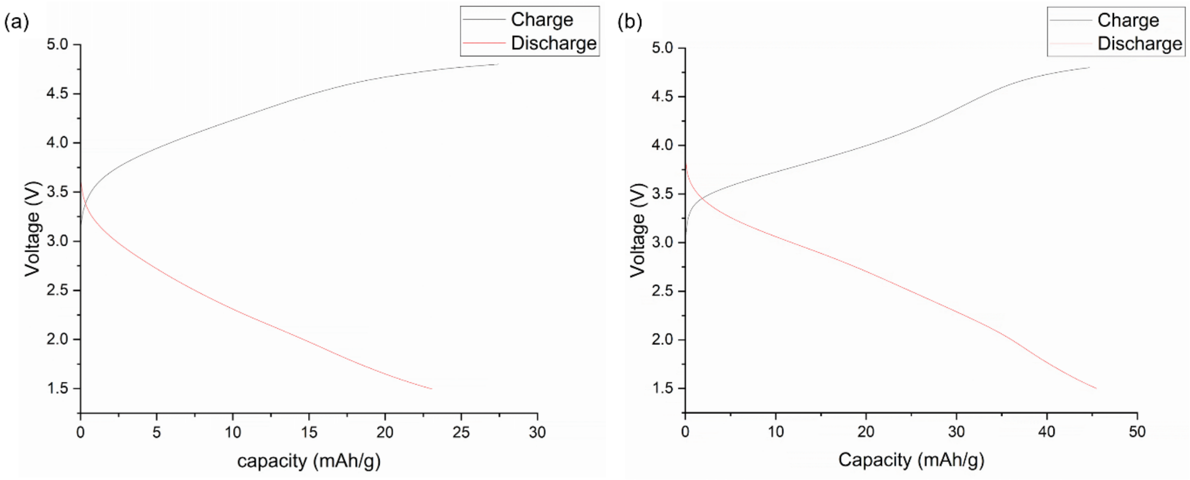

Figure S4: charging and discharge curve of LMTO-B, LMTOF-B between 1.5-4.8 V at 0.5 C.

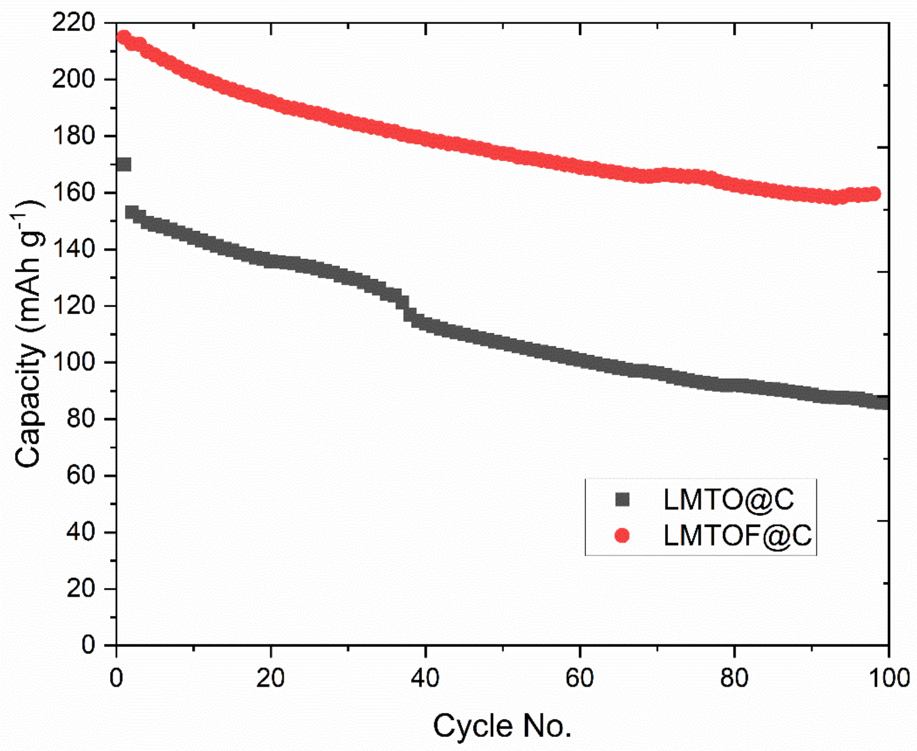

Figure S5: Cycling performance of LMTO@C, LMTOF@C between 1.5-4.8 V at 0.5 C.
